# Circulating miRNAs in extracellular vesicles related to treatment response in patients with idiopathic membranous nephropathy

**DOI:** 10.1186/s12967-022-03430-7

**Published:** 2022-05-14

**Authors:** In O. Sun, Yun-Ui Bae, Haekyung Lee, Hyoungnae Kim, Jin Seok Jeon, Hyunjin Noh, Jong-Soo Choi, Kyung-Oh Doh, Soon Hyo Kwon

**Affiliations:** 1 Division of Nephrology, Department of Internal Medicine, Presbyterian Medical Center, Jeonju, Republic of Korea; 2 Department of Internal Medicine, Keimyung University Dongsan Hospital, Keimyung University School of Medicine, Daegu, Republic of Korea; 3 Division of Nephrology, Soonchunhyang University Seoul Hospital, 59 Daesagwan-ro, Youngsan-gu, Seoul, 04401 Republic of Korea; 4 Department of Physiology, College of Medicine, Yeungnam University, Daegu, 42415 Republic of Korea

**Keywords:** Extracellular vesicles, microRNAs, Glomerulonephritis, Treatment outcome

## Abstract

**Background:**

Extracellular vesicle (EV)-microRNAs (miRNAs) are potential biomarkers for various renal diseases. This study attempted to identify the circulating EV-miRNA signature not only for discriminating idiopathic membranous nephropathy (IMN) from idiopathic nephrotic syndrome (INS), but also to predict the treatment response of patients with IMN.

**Methods:**

We prospectively enrolled 60 participants, including those with IMN (n = 19) and INS (n = 21) and healthy volunteers (HVs; n = 20) in this study. Using RNA sequencing, we assessed the serum EV-miRNA profiles of all participants. To identify the EV-miRNAs predictive of treatment response in IMN, we also analyzed EV-miRNAs among patients with IMN with and without clinical remission.

**Results:**

The expression levels of 3 miRNAs differed between IMN patients, INS patients and HVs. In addition, compared to HVs, RNA sequencing revealed differential expression of 77 and 44 EV-miRNAs in patients with IMN without and with remission, respectively. We also identified statistically significant (|fold change ≥ 2, p < 0.05) differences in the expression levels of 23 miRNAs in IMN without remission. Biological pathway analysis of miRNAs in IMN without remission indicated that they are likely involved in various pathways, including renal fibrosis.

**Conclusion:**

Our study identified EV-miRNAs associated with IMN as well as those associations with therapeutic response. Therefore, these circulating EV-miRNAs may be used as potential markers for the diagnosis and prediction of treatment response in patients with IMN.

**Supplementary Information:**

The online version contains supplementary material available at 10.1186/s12967-022-03430-7.

## Background

Nephrotic syndrome is associated with heavy proteinuria and peripheral edema [1]. Idiopathic membranous nephropathy (IMN) is among the most common etiologies of primary nephrotic syndrome and its incidence has been rising in recent years [2]. Most patients with IMN or idiopathic nephrotic syndrome (INS) present with marked proteinuria and peripheral edema [1, 3]. Many observations support that a circulating factor may be responsible for some forms of nephrotic syndrome, including IMN [3–5]. Although renal biopsy is the gold standard for differentiating glomerulonephritis from nephrotic syndrome, it is an invasive procedure and can sometimes be dangerous in patients taking antiplatelet agents or anticoagulation medications. Therefore, in cases where there is a relative contraindication to renal biopsy, a serology-based approach to diagnosing IMN has been suggested in a previous study [6]. Studies have shown that the serum anti-phospholipase A2 receptor (PLA2R) antibody can be a potential biomarker for diagnosing, measuring disease activity, and predicting response to treatment in IMN [7–9]. Lower anti-PLA2R titers are associated with high rates of spontaneous remission in IMN, thus favoring conservative therapy, while declining anti-PLA2R titer levels after treatment predict clinical response to rituximab treatment [7]. However, the sensitivity of anti-PLA2R titer for the detection of IMN is low (between 52 and 78%) [10–13], and 20–30% of patients test negative for serum anti-PLA2R antibodies [14–16].

Extracellular vesicles (EVs) contain various molecules, including proteins, lipids, DNA, mRNA, and microRNA (miRNA), originating from the cell, and among these molecules, miRNAs have attracted the most attention since they can stably exist in various body fluids and play regulatory roles in gene expression [17, 18]. EV-miRNAs appear to be more stable than free miRNAs, as EVs seem to protect and increase the stability of miRNAs [19]. Recently, EV-miRNAs were reported to be more useful than free miRNAs for the detection of acute kidney injury (AKI) [20]. In addition to AKI, some studies have shown the potential of EV-miRNAs to server as biomarkers in glomerulonephritis, such as for lupus nephritis [21, 22]. Previous EV studies in renal diseases have usually focused on urinary EVs [23, 24], whereas some recent reports have indicated a unique profile of circulating EV-miRNAs for nephrotic syndrome [25–27]. These results suggest that circulating miRNAs could be used as biomarkers for nephrotic syndrome**.** We also found that EV-miRNA profiles differ between the patients with diabetic nephropathy and those without diabetic nephropathy [28]. However, there is limited data showing the potential role of miRNAs as diagnostic, prognostic, and therapeutic biomarkers for IMN.

In this study, to identify IMN-specific miRNAs at the time of kidney biopsy, we compared the EV-miRNA profiles of patients with IMN and other cohorts, including healthy volunteers (HVs) and patients with INS, using RNA sequencing. Furthermore, we attempted to identify circulating EV-miRNAs predictive of treatment response in patients with IMN by comparing EV-miRNA profiles in patients with and without clinical remission during treatment.

## Materials and methods

### Participants and data collection

We included 40 age- and sex-matched patients with IMN and INS and 20 HVs from a prospective glomerular disease cohort. In the present study, INS included focal segmental glomerular sclerosis and minimal change disease, which were defined by the association of the clinical features of nephrotic syndrome with renal biopsy findings of diffuse foot process effacement using electron microscopy [29]. Patients with glomerulonephritis, who were diagnosed between January 2015 and Jun 2020, were enrolled from Soonchunhyang University Hospital and Presbyterian Medical Center. Patients with secondary causes of MN, such as lupus or malignancy, were excluded. This study was approved by the institutional review board of Soonchunhyang University (IRB No. 2016-01-002-007). Written informed consent was obtained from all the participants.

Remissions were defined according to the 2012 Kidney Disease: Improving Global Outcomes guidelines. Among patients with IMN, complete remission was defined as a reduction in proteinuria to 0.3 g/day. Partial remission was defined as a reduction in proteinuria to between 0.3 and 3.5 g/day (with at least 50% reduction versus baseline). Composite remission included either complete remission within 1 year after renal biopsy or partial remission with less than 2.5 g of proteinuria for 2 years following pathologic diagnosis. A refractory response was defined as the absence of composite remission during the follow-up period. Therefore, a total of 19 patients with IMN were divided into two groups: a well- responding (IMN-W) and refractory (IMN-R) group, based on the achievement of composite remission. Anti-PLA2R antibody was measured by ELISA method (EUROIMMUN AG, Lubeck, Germany) using serum sample collected on kidney biopsy.

During the follow-up period, treatment decisions for the enrolled patients were made by the treating nephrologist. The most common reasons for initiating immunosuppressive therapy were patient characteristics (proteinuria, renal function, etc.) that were not properly controlled, and nephrologist clinical judgement.

### Serum EV RNA isolation and assessment

RNA sequencing was conducted as previously described [30]. Briefly, circulating EVs were isolated from the serum (1000 μL) using the ExoQuick isolation agent (System Bioscience, Palo Alto, CA, USA), according to the manufacturer’s guidelines. Supernatants obtained after centrifugation (3000 ×*g* for 15 min) of the serum samples were mixed with ExoQuick reagent and incubated for 30 min at 4 °C. After another centrifugation at 1500 ×*g* for 30 min, the supernatant was aspirated, and the pellet was retained. After resuspension of the pellet in sterile phosphate-buffered saline (200 μL), RNA was extracted using the miRNeasy Mini Kit (Qiagen, Hilden, Germany). All processes involving the suspension of exosomes were conducted according to the manufacturer’s guidelines. After RNA extraction, purified RNA was eluted in RNase-free water (20 μL). The purified RNA was analyzed using an Agilent Bioanalyzer 2100 with an RNA Pico Chip and Small RNA Chip to examine the size distribution of EV RNAs (Agilent Technologies, Santa Clara, CA, USA).

### Characterization of EVs by cluster of differentiation 63 (CD63) detection

CD63 levels in circulating EVs were measured using the Exo-enzyme-linked immunosorbent assay (ELISA)-ULTRA CD63 kit (System Biosciences, Palo Alto, CA, USA), according to the manufacturer’s protocol.

### Western blot analysis

Each sample were electrophoresed on SDS-PAGE gels and were transferred to nitrocellulose membranes. The membranes were probed with specific antibodies as follows; anti-CD9 (Abcam, Cambridge, MA, USA) and anti-GM-130 (Abcam). The membranes were incubated with horseradish peroxidase-coupled secondary antibody (Sigma). Following washing with TBS-T, the bound antibody was detected by enhanced chemiluminescence (Amersham, Buckinghamshire, UK).

### Transmission electron microscopy (TEM)

This protocol was performed as described by Thery et al. and Rikkert et al. [31, 32]. A droplet of exosome solution was placed on Para film, and a Formva-carbon-coated nickel grid (200 meshes, TED PELLA, USA) was floated on the drop to absorb the sample at room temperature. After 10 min, the exosomes were fixed with 2.5% glutaraldehyde and stained with 1% uranyl acetate. The sample was washed with distilled water and dried in the dark. The grid was observed using an electron microscope operating at 75 kV (H-7000B; Hitachi, Tokyo, Japan).

### Exosome physicochemical properties

A Nano-ZS Zetasizer (Malvern Inc., UK) was used to estimate the particle size. The samples were diluted ten times with distilled water and particle size was measured three times in a set of 50 repetitions using disposable cuvettes (DTS1070; Malvern Inc., Worcestershire, UK) and analyzed using the Zetasizer software (version 7.11).

### cDNA library preparation and small RNA sequencing

The samples were processed to produce exosomal RNA (10 ng) as an input for each library. Small RNA libraries were constructed using a SMARTer smRNA-Seq Kit for Illumina (Takara Bio, Shiga, Japan), according to the manufacturer’s guidelines. Sequencing libraries were constructed by polyadenylation, cDNA synthesis, and polymerase chain reaction (PCR) amplification.

The libraries were gel-purified and validated by assessing their size, purity, and concentration using an Agilent Bioanalyzer. The libraries were quantified using quantitative PCR (qPCR), according to the qPCR Quantification Protocol Guide (KAPA Library Quantification Kits for Illumina® Sequencing Platforms). We assessed the quality of the libraries using TapeStation D1000 ScreenTape (Agilent Technologies, Waldbronn, Germany). Equimolar amounts of libraries were pooled and sequenced on an Illumina HiSeq 2500 instrument (Illumina, San Diego, CA, USA) to generate 51 base reads. Image decomposition and quality value calculations were performed using modules in the Illumina pipeline. All procedures for next-generation sequencing (NGS) analysis were performed at Macrogen (Seoul, Korea).

### Analysis of RNA sequencing data and proportions of miRNAs

Following sequence alignment, known and novel miRNAs were identified using the miRDeep2 algorithm. Prior to sequence alignment, we retrieved the *Homo sapiens* reference genome release hg19 from the UCSC Genome Browser, which was indexed using Bowtie (1.1.2), a program for aligning experimental and reference sequences. The reads were then aligned to the mature and precursor *H. sapiens* miRNAs obtained using miRBase 21. Uniquely clustered reads were sequentially aligned to the reference genome using miRBase 21 and the non-coding RNA database Rfam 9.1 to identify known miRNAs and other types of RNAs, respectively.

### Analysis of miRNA expression levels

The raw data (reads for each miRNA) were normalized to the relative log expression using DESeq2. For preprocessing, miRNAs absent from more than 50% of all samples were excluded, leaving only mature miRNAs for analysis. We added 1 to the normalized read count of the filtered miRNAs to facilitate log_2_ transformation and draw a correlation plot. For each miRNA, the base mean and log-fold changes were calculated between the groups. We conducted a statistical hypothesis test to compare the groups using the negative binomial Wald test in DESeq2. miRNAs differentially expressed between the two groups were defined as having a |fold change|≥ 2 and a false discovery rate (FDR)-adjusted p-value of < 0.05. We also performed hierarchical clustering analysis using complete linkage and Euclidean distance as measures of similarity to display the expression patterns of the differentially expressed miRNAs that satisfied the criteria of a |fold change|≥ 2 and an FDR-adjusted p-value of < 0.05. All data analyses and visualization of the differentially expressed genes were performed using R 3.3.1 (www.r-project.org).

### Identification of miRNA target genes and their molecular pathways

We uploaded miRNAs that were differentially expressed in the HVs and patients with IMN-W and IMN-R into commonly used analysis programs, such as DIANA-miRPath and miRSystem, for further analyses. The DIANA-miRPath v.3.0 database used DIANA-microT-CDS and TargetScan 6.2 to analyze miRNA-gene interactions. The database schema incorporated the Kyoto Encyclopedia of Genes and Genomes (KEGG) pathways, Gene Ontology (GO), and GO slim annotations. Gene and miRNA annotations were derived from the Ensembl and miRBase databases, respectively.

### Statistical analysis

Continuous variables with normal distributions are expressed as the mean ± standard deviation; variables without a normal distribution are expressed as medians with interquartile ranges. The *t*-test was used to analyze the statistical significance of the differences between continuous variables, and the chi-square test was used for categorical variables to compare the baseline characteristics between HVs and patients with IMN. The IMN group was further divided into two groups, and continuous variables were compared among the three subgroups (HVs vs. IMN-W vs. IMN-R) using the Kruskal–Wallis multiple comparison test. Receiver operating characteristics (ROC) analysis was used to calculate the area under the curve (AUC) for each miRNA for the diagnosis and prediction of treatment of response in IMN patients. Statistical significance was set at P < 0.05. Statistical analysis was performed using SPSS (version 22.0; IBM Corp., Armonk, NY, USA).

## Results

### Baseline clinical characteristics

The participants included 34 (56%) men, with a mean age of 55 years (range, 25–85 years). The baseline characteristics of the participants are presented in Table 1. HVs had no history of hypertension, diabetes, or medication use. When we compared the clinical characteristics between the IMN and INS groups, we found no differences in renal function or proteinuria. Similarly, no differences in baseline characteristics were observed between the IMN-W and IMN-R groups. Follow-up proteinuria in the IMN-R group was greater than that in the IMN-W group (490 vs. 5458 mg/day, p = 0.007) (Table 2). Within the follow-up period, immunosuppressants, including steroids and cyclosporine, were prescribed to six (67%) and five (50%) patients in the IMN-W and in IMN-R groups, respectively. Of the immunosuppressive drugs, combination of cyclosporine and steroid is the most common regimen in both group.

**Table 1:** Comparison of baseline characteristics among three groups

	HV (n = 20)	INS (n = 21)	IMN (n = 19)	P-value
Age	53 ± 11	56 ± 16	57 ± 14	0.653
Male, n (%)	11 (55)	12 (57)	11 (58)	0.488
DM, n (%)	0 (0)	16 (76)	11 (58)	0.185
Hypertension, n (%)	0 (0)	11 (52)	11 (58)	0.488
Hemoglobin (mg/dl)	13.9 ± 1.2	13.5 ± 2.3	13.0 ± 1.8	0.301
Serum albumin (mg/dl)	4.6 ± 0.2^a^	2.5 ± 1.0^b^	2.4 ± 0.5^b^	< 0.001
Triglyceride (mg/dl)	116 ± 53^a^	314 ± 264^b^	300 ± 199^b^	< 0.001
eGFR (ml/min/1.73m^2^)	83 ± 16	71 ± 33	79 ± 22	0.268
24 h-proteinuria (mg/day)	85 ± 54^a^	9181 ± 5920^b^	7141 ± 4185^b^	< 0.001

The same letters (a or b, respectively) indicate non-significant difference between groups based on Kruskal Wallis multiple comparison test

**Table 2:** Comparison of baseline characteristics between two groups

	IMN-W (n = 9)	IMN-R (n = 10)	P-value
Age	56 (37–74)	59 (36–84)	0.702
Male, n (%)	5 (56)	6 (60)	0.605
DM, n (%)	6 (67)	5 (50)	0.395
Hypertension, n (%)	7 (78)	4 (40)	0.115
Hemoglobin (mg/dl)	12.8 (11.1–15.1)	12.5 (10.0–18.0)	0.447
Serum albumin (mg/dl)	2.2 (1.6–3.6)	2.3 (1.5–3.0)	0.968
Triglyceride (mg/dl)	242 (146–871)	209 (123–611)	0.447
eGFR (ml/min/1.73m^2^)	89 (42–110)	75 (48–109)	0.549
24 h-proteinuria, baseline	6462 (110–17,296)	8084 (2148–13,044)	0.356
Anti-PLA2R Ab	0.6 (1–214)	61.2 (1–566)	0.258
Treatment regimen			
ACEi or ARB	7 (78)	10 (100)	0.211
Immunosuppressive drugs	6 (67)	5 (50)	0.395
CsA + Steroid	3	3	
CTX + Steroid	2	1	
Tac + Steroid	1	1	

*eGFR* estimated glomerular filtration rate, *ACEi* angiotensin converting enzyme inhibitor, *ARB* angiotensin receptor blocker, *CsA* cyclosporine, *CTX* cyclophosphamide, *Tac* tacrolimus

### Characterization of circulating EVs and small RNA composition changes

**Fig. 1:**
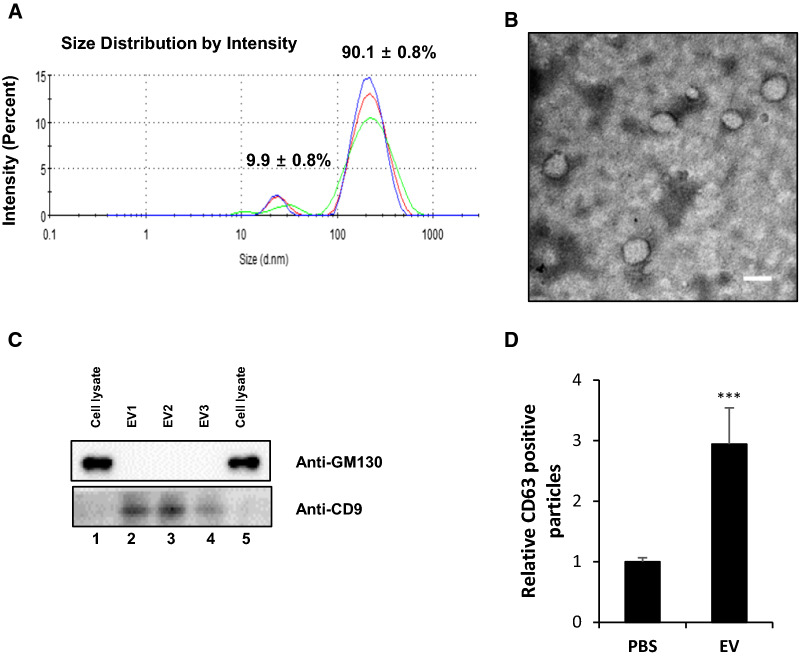
Isolated extracellular vesicles (EVs) from the serum samples of patients. **A** Diagram of the observed peaks of nanoparticles in three patient serum EVs by dynamic light scattering (DLS) using Nano-ZS Zetasizer. **B** Transmission electron microscopy showing membranous vesicles of purified serum EVs. Scale bar, 200 nm. **C** Western blot analysis of EVs using positive marker as CD9 and negative marker as GM-130. **D** Cluster of differentiation 63 (CD63) expression levels in the serum-derived EVs as determined by the enzyme-linked immunosorbent assay (ELISA). Bars represent mean ± SD of three EVs from different samples

Circulating EVs were isolated and characterized. The median diameter of the EVs measured by dynamic light scattering was 179 ± 73.5 nm (Fig. 1A) and 161.3 ± 29.2 nm when measured by electron microscopy (Fig. 1B). Western blotting verified that CD9, well-known EV marker protein, was present (Fig. 1C). The absence of GM-130, a Golgi marker, excluded the potential contamination with components with cellular vesicular structures (Fig. 1C) [33]. The levels of CD63, an exosome marker, were higher in the isolated EV samples (Fig. 1DC). EV RNA was isolated and analyzed quality of RNA by bioanalyzer for NGS (Additional file 1: Fig. S1). To examine the EV small RNA composition, we conducted NGS followed by mapping to each small RNA reference database. Using NGS, we identified EV small RNAs, including miRNAs, small nuclear RNAs (snRNAs), small nucleolar RNAs (snoRNAs), and transfer RNAs. The proportion of small RNAs that were miRNAs was lower in the IMN-R group than in the HVs and IMN-W group, whereas the proportion that were snoRNAs and snRNAs was higher in the IMN-R group than the HVs and IMN-W groups (Additional file 1: Fig. S2).

### Comparison of EV-miRNAs among patients with INS and IMN, and HVs

**Fig. 2:**
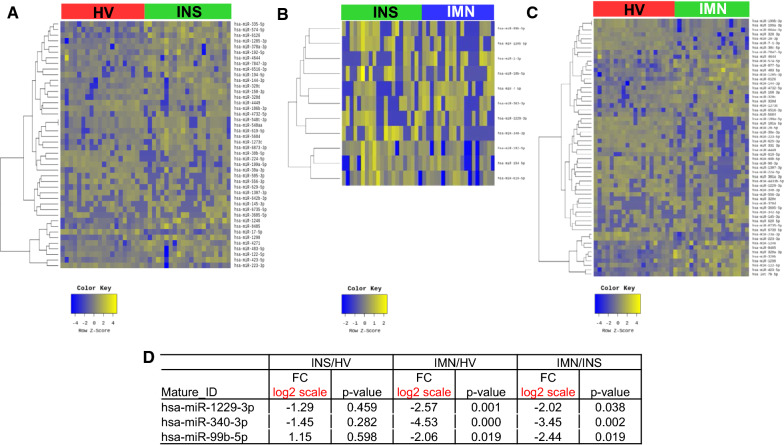
EVs-microRNAs (miRNAs) from patients with idiopathic nephrotic syndrome (INS), idiopathic membranous nephropathy (IMN), and healthy volunteers (HVs). **A** Heat map showing z-scores of EVs-miRNAs from HVs (n = 20) and patients with INS (n = 21), with 33 upregulated (yellow) and 11 downregulated (blue) miRNAs. **B** Heat map showing z-scores of EVs-miRNAs from patients with INS (n = 21) and IMN (n = 19), with 3 upregulated (yellow) and 8 downregulated (blue) miRNAs. **C** Heat map showing z-scores of EVs-miRNAs from HVs (n = 20) and patients with IMN (n = 19) with 31 upregulated (yellow) and 27 downregulated (blue) miRNAs. **D** Fold change (FC) and p-values of three miRNAs, whose expression levels were specifically up- or down-regulated in patients with IMN compared to HVs and patients with INS

After RNA sequencing, we identified 33 miRNAs that were upregulated and 11 that were downregulated in patients with INS when compared to HVs (Fig. 2A). We also found three miRNAs that were upregulated and eight that were downregulated in patients with IMN when compared to patients with INS (Fig. 2B). There were 31 miRNAs that were upregulated and 27 that were downregulated in patients with IMN when compared to the HVs (Fig. 2C). Among these miRNAs, we found three miRNAs (miRNA-1229-3p, miRNA-340-3p, and miRNA-99b-5p), whose levels were significantly up- or downregulated in patients with IMN compared to HVs and patients with INS (Fig. 2D). The area under the ROC for each miRNA was shown (Additional file 1: Fig. S3). In addition, the expression levels of two miRNAs (miRNA-192-5p and miRNA-194-5p) in circulating EVs were significantly different in patients with INS compared to those in HVs and IMN groups (Additional file 1: Fig. S4).

### Identification and analysis of differentially expressed miRNAs between IMN-W and IMN-R

**Fig. 3:**
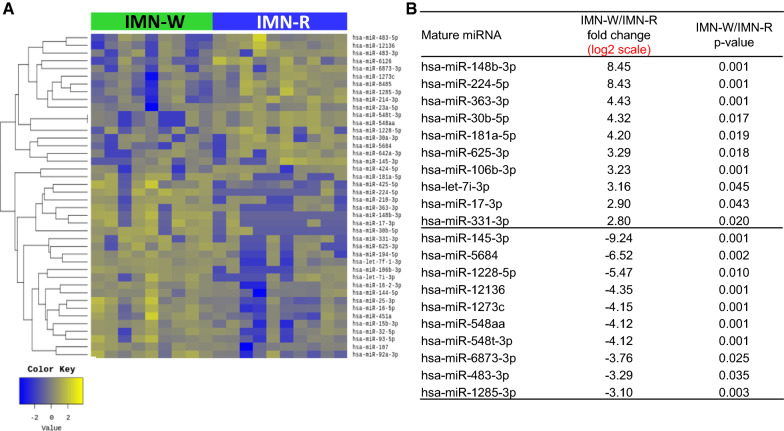
Extracellular vesicle (EV-miRNAs from patients with idiopathic membranous nephropathy (IMN) with (IMN-W, n = 9) and without (IMN-R, n = 10) clinical remission. **A** Heat map showing z-scores of EVs-miRNAs from patients with IMN, with or without clinical remission, with 24 upregulated (yellow) and 18 downregulated (blue) miRNAs. **B** Fold change (FC) and p-values of top 10 miRNAs showed differential expression in patients with IMN-W and IMN-R

**Fig. 4:**
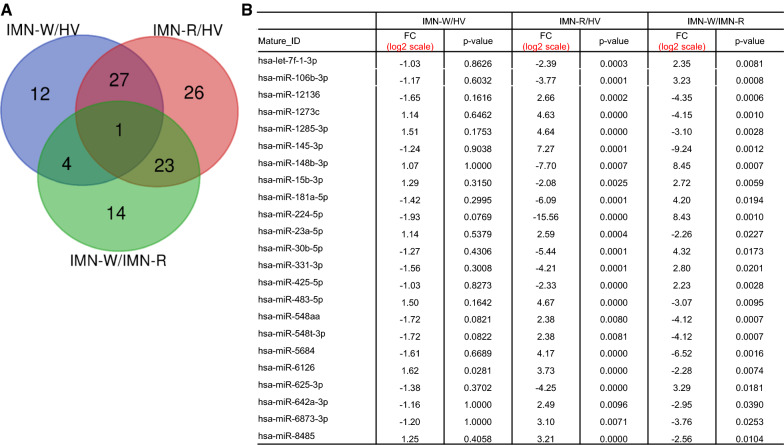
Identification of idiopathic membranous nephropathy without clinical remission (INM-R)-specific EVs-miRNAs. **A** Venn diagram of overlapping miRNAs among the three datasets shows 23 miRNAs expressed in patients with IMN-R. **B** Fold change (FC) and p-values of 23 miRNAs, whose expression levels were up- or down-regulated in patients with IMN-R compared to HVs and IMN-W

**Fig. 5:**
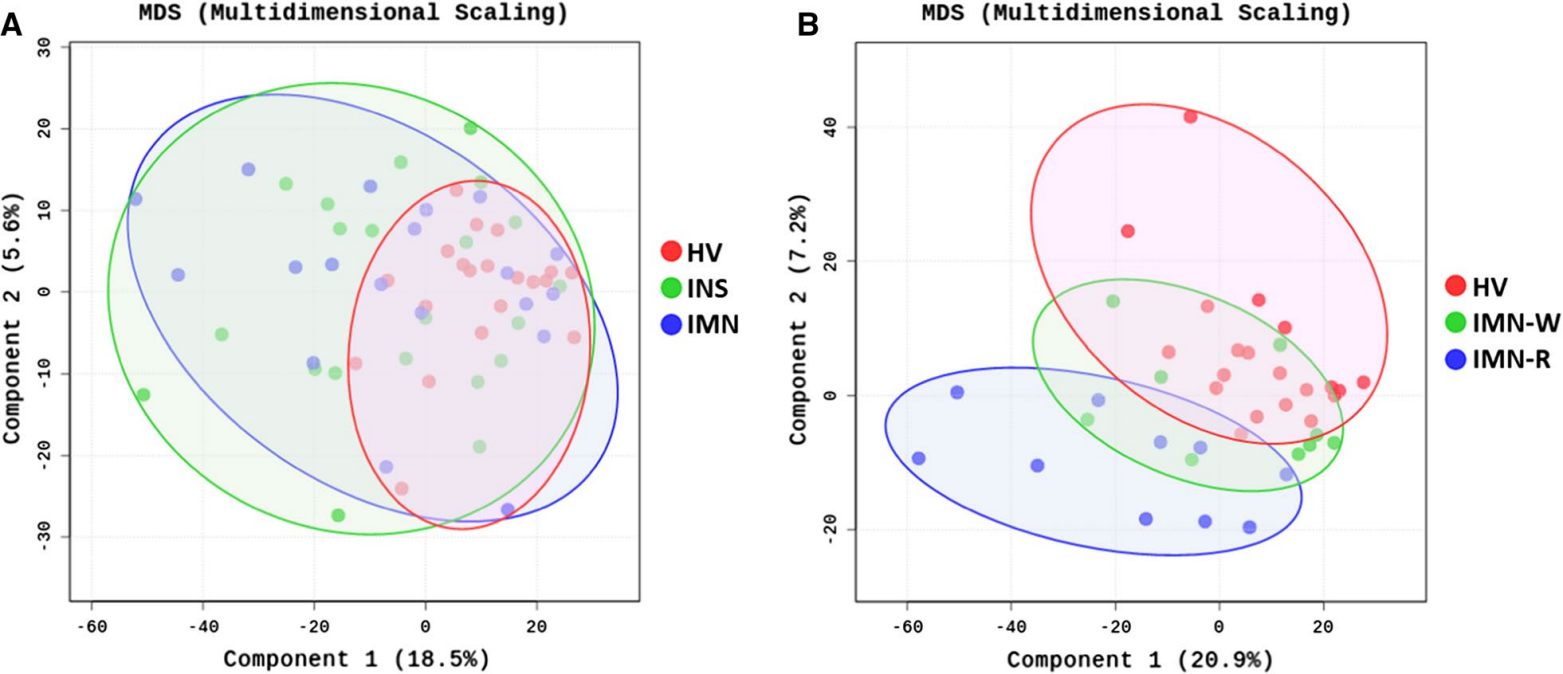
Principle component analysis (PCA) of serum-derived EVs. **A** Results of PCA using serum-derived EVs from HVs, IMN, and INS. **B** Results of PCA using serum-derived EVs from HVs, IMN-W, and IMN-R

Furthermore, we found 21 upregulated and 23 downregulated miRNAs in patients with IMN-W when compared to HVs (Additional file 1: Fig. S5). We also found 37 upregulated and 40 downregulated miRNAs in patients with IMN-R when compared to HVs (Additional file 1: Fig. S6). Between the two groups of patients with IMN, there were 24 upregulated and 18 downregulated miRNAs in patients with IMN-R when compared to patients with IMN-W (Fig. 3). Among the differentially expressed miRNAs, we identified 23 that were expressed in patients with IMN-R (Fig. 4). The results of principle component analysis (PCA) using serum-derived EVs from the HV, IMN-W, and IMN-R groups are shown in Fig. 5. We evaluated the predicted biological pathways associated with these miRNAs using the miRSystem. The possible pathways associated with IMN-R are presented in Table 3. miRNAs involved in renal fibrosis are listed in Table 4.

**Table 3:** Top list of possible canonical pathways associated with 23 miRNAs which differentially expressed in patients with IMN-R

KEGG Pathway	Total genes in pathway	miRNAs	p-value
Proteoglycans in cancer	95	17	1.25E−07
TGF-beta signaling pathway	46	15	2.26E−07
Long-term depression	35	15	2.26E−07
Hippo signaling pathway	73	16	2.26E−07
Prion diseases	12	11	7.45E−07
Transcriptional misregulation in cancer	84	18	1.02E−06
Glioma	38	15	2.42E−06
Renal cell carcinoma	43	15	4.93E−06
Chronic myeloid leukemia	45	15	4.99E−06
FoxO signaling pathway	73	16	6.65E−06
Axon guidance	68	15	1.45E−05
Acute myeloid leukemia	36	14	1.66E−05
Pathways in cancer	178	17	1.67E−05
Rap1 signaling pathway	105	18	2.48E−05
Viral carcinogenesis	83	18	2.60E−05
Adherens junction	44	16	4.85E−05
Colorectal cancer	40	15	9.85E−05
Signaling pathways regulating pluripotency of stem cells	69	17	0.000102
Focal adhesion	102	15	0.000137
Non-small lung cancer	32	15	0.000151

**Table 4:** Relationship between renal fibrosis pathway and miRNAs associated with IMN-R

miRNAs	TGF-beta	Hippo	FOXO	Rap1	Proteoglycan
hsa-let-7f-1-3p	●	●	●	●	●
hsa-miR-1273c	▬	▬	▬	●	●
hsa-miR-1285-3p	●	●	●	●	●
hsa-miR-148b-3p	●	●	●	●	●
hsa-miR-15b-3p	▬	●	●	●	●
hsa-miR-181a-5p	●	●	●	●	●
hsa-miR-224-5p	●	●	●	●	●
hsa-miR-23a-5p	●	●	▬	●	●
hsa-miR-30b-5p	●	●	●	●	●
hsa-miR-425-5p	●	●	●	●	●
hsa-miR-483-5p	▬	▬	▬	●	▬
hsa-miR-548aa	●	●	●	●	●
hsa-miR-548t-3p	●	●	●	●	●
hsa-miR-5684	▬	▬	●	▬	▬
hsa-miR-6126	●	●	●	●	●
hsa-miR-625-3p	●	●	●	●	●
hsa-miR-642a-3p	●	●	●	●	●
hsa-miR-6873-3p	●	●	●	●	●
hsa-miR-8485	●	●	●	●	●

● Presence

▬ Absence

### Correlation between EV-miRNAs and clinical parameters

Of the EV-miRNAs that were differentially expressed in patients with IMN-R, the expression levels of miRNA-1285-3p, miRNA-23a-5p, miRNA-483-5p, and miRNA-6126 were found to be directly correlated with proteinuria on kidney biopsy (Table 5). Meanwhile, a negative correlation was observed between renal function and expression of miRNAs, such as miRNA-12136 and miRNA-483-5p. A significant association with the anti-PLA2R antibody was found only for miRNA-23a-5p.

**Table 5:** Correlation of differentially expressed miRNAs in IMN-W and –R with clinical parameters

	GFR		24-Proteinuria		Anti-PLA2R Ab	
	r	p	r	p	r	p
IMN-W miRNAs						
hsa-miR-107	0.017	0.919	0.255	0.122	− 0.230	0.374
hsa-miR-32-5p	0.188	0.288	0.205	0.244	0.217	0.456
hsa-miR-424-5p	0.077	0.651	− 0.187	0.268	0.275	0.302
hsa-miR-451a	0.217	0.185	0.070	0.674	− 0.316	0.201
IMN-R miRNAs						
hsa-let-7f-1-3p	0.075	0.665	− **0.370**	**0.027**	0.125	0.656
hsa-miR-106-3p	− 0.097	0.592	− 0.263	0.139	0.241	0.427
hsa-miR-12136	− **0.460**	**0.003**	0.043	0.793	0.233	0.353
hsa-miR-1285-3p	− 0.251	0.129	**0.447**	**0.005**	0.391	0.108
hsa-miR-15b-3p	0.098	0.558	− 0.252	0.127	− 0.267	0.300
hsa-miR-23a-5p	-0.185	0.272	**0.375**	**0.022**	**0.616**	**0.008**
hsa-miR-425-5p	0.163	0.321	− **0.366**	**0.022**	− 0.006	0.981
hsa-miR-483-5p	− **0.366**	**0.022**	**0.372**	**0.020**	0.274	0.270
hsa-miR-6126	− 0.228	0.163	**0.504**	**0.001**	0.157	0.535
hsa-miR-625-3p	0.005	0.979	− 0.302	0.093	− 0.479	0.098
hsa-miR-8485	− 0.291	0.073	0.289	0.074	0.390	0.110

Statistically significant data are shown in bold

## Discussion

In this study, we conducted RNA sequencing to examine the circulating EV-miRNA profiles of patients with IMN and compared them with those of patients with INS and HVs. We identified IMN-specific EV-miRNAs compared to those in HVs or INS subjects. Furthermore, we found that EVs-miRNAs were associated with treatment response in patients with IMN, which will be helpful for clinicians to predict the prognosis of these patients.

Differentially-expressed circulating miRNAs have been found in patients with various glomerular diseases, such as IgA nephropathy and lupus nephritis [34, 35]. Significant differences in the expression profiles of urinary and circulating exosomal miRNAs have also been observed between HVs and patients with IMN [36, 37]. A previous study suggested there is miRNA dysregulation in IMN, in which the differential expression of six miRNAs (upregulation of miR-152 and -15 and downregulation of miR-82, -98, -89, and -84) was observed in patients with IMN when compared to HVs [38]. These miRNAs did not overlap with those identified in the present study. This difference might be due to the source of miRNAs, since the authors of the previous study analyzed miRNAs from peripheral blood lymphocytes. In the present study, we identified three IMN-specific EVs-miRNAs, which might be helpful in discriminating patients with IMN from those with INS or without nephrotic syndrome. Therefore, this study demonstrates the potential of EV-miRNAs as biomarkers for IMN diagnosis.

In addition to these miRNAs, we identified EVs-miRNAs that are helpful in predicting treatment response in patients with IMN. To our knowledge, this is the first study of EV-miRNAs for predicting treatment response in patients with IMN. The prediction of clinical remission in patients with IMN is important for nephrologists because complete or partial remission is associated with good kidney survival [39, 40]. When a refractory case is anticipated with conventional therapy, the clinician may be able to come up with alternative treatment options to avoid the adverse effects of immunosuppressive drugs. In our study, we identified 23 miRNAs that were differentially expressed in patients with IMN-R which were distinct from those in patients with IMN-W. Interestingly, such pathways seem to be associated with cancers. Although the causality link between malignancy and MN remain unsolved, MN is the most common type of malignancy-associated glomerular lesions. Recently, von Haxthausen et al. reported no differences in antigen-specific IgG subclasses between IMN and malignancy-associated MN [41], suggesting a similar pathogenesis between idiopathic and malignancy-associated MN. Therefore, we believe that circulating EVs-miRNAs could be used as non-invasive markers for predicting clinical remission in patients with IMN during treatment.

Of the possible pathways associated with treatment response in patients with IMN in the present study, the transforming growth factor-β (TGF-β) signaling pathway seems to be associated with predicting the treatment response based on our data. Renal fibrosis is the usual final outcome of excessive accumulation of extracellular matrix, and TGF-β plays an important role in tissue fibrosis by upregulating matrix protein synthesis and inhibiting matrix degradation [42]. Previous studies have shown that urinary TGF-β1 levels are elevated during active disease and correlate with histological characteristics and proteinuria [43–45]. In patients with MN, higher initial urinary TGF-β1 levels are associated with persistent nephrotic syndrome and kidney function decline at 12 months [44, 46]. Of the 15 miRNAs associated with the TGF-β pathway in our study, the levels of let-7f and miR-23a were associated with baseline proteinuria on kidney biopsy, which is known to be a predictor of IMN [47, 48]. In addition, EV-miR-23a levels directly correlated with anti-PLA2R antibody concentrations. These findings are consistent with previous experimental results. Overexpression of miR-23a has been reported to repress renal cell viability and proliferation by suppressing the heat shock protein 70 [49]. In addition, upregulation of let-7f in diabetic mice is helpful in relieving podocyte injury in diabetic nephropathy [50]. Interestingly, proteoglycans, which are a family of highly glycosylated proteins mainly involved in tissue organization, seems to be associated with differentially expressed miRNAs in patients with IMN-R in this study. Such finding might be due to the inhibitory influence on TGF-β signaling pathway [51].

Besides the TGF-β pathway, based on the identified miRNAs in our study, the Hippo signaling, Forkhead homobox type O (FoxO), and Rap1 pathways are analyzed to be associated with renal fibrosis in patients with IMN-R. The Hippo signaling pathway not only participates in the crosstalk with other signaling pathways, including the TGF-β and Wnt/β-catenin signaling pathways, but also plays a role in the pathogenesis of renal tubulointerstitial fibrosis [52, 53]. The Rap1 pathway influences renal fibrosis via regulation of the cyclic adenosine 3′,5'-monophosphate signaling pathway [54]. In this study, some miRNAs, such as let-7f and miR-23a, were also found to be associated with the FoxO and Hippo pathways, similar to the TGF-β pathway. Previous studies have also reported such a relationship in other diseases [55, 56]. Intriguingly, PCA of serum-derived EVs from HVs and the IMN, and INS groups revealed a relatively poor demarcation. However, PCA revealed some overlap between the serum-derived EVs from HVs and those from patients with IMN-W, with a fully distinct miRNA profile for serum-derived EVs from patients with IMN-R. Although these results might imply that fibrotic changes in IMN-R are important factors affecting treatment response, further studies are needed to investigate the pathological mechanism for predicting the clinical responses of patients with IMN. Our study had some limitations. First, the number of enrolled participants was relatively small; therefore, larger prospective randomized controlled trials are needed to confirm our results. Next, EV RNA was isolated using commercial kits; however, isolation using other methods may yield different results. To date, there is no gold standard method for EV isolation. Finally, we could not validate our findings in different cohorts, and this can be taken up in future studies.

## Conclusions

Our study revealed circulating EV-miRNAs that can discriminate patients with IMN from HVs and those with INS. Furthermore, we identified circulating EV-miRNAs that are associated with clinical remission in patients with IMN, which may be used as surrogate markers for predicting clinical remission in these patients during treatment. However, further studies are needed to confirm the utility of EV-miRNAs in diagnosing IMN and predicting clinical remission in patients with IMN.

## Supplementary Information

**Additional file 1:** **Figure S1.** Bioanalyzer analysis of the size distribution of RNA from EVs. (A) The Pico 6000 chip analyzed total EV RNA (< 6000 nucleotides). (B) The Small RNA chip analyzed small RNA of EV (< 200 nucleotides).** Figure S2.** Small RNA composition differences in circulating extracellular vesicles (EVs) by RNA sequencing. *P < 0.05 vs patients with idiopathic membranous nephropathy with clinical remission (IMN-W), **†**P < 0.05 vs healthy volunteers (HVs). **Figure S3.** Performance of IMN-specific EV-miRNAs for the discrimination of IMN and INS. Receiver operating curves show the distinguishing performance of three miRNAs (miRNA-1229-3p, miRNA-340-3p, and miRNA-99b-5p) that obtained from 19 patients with IMN and 21 patients with INS. **Figure S4.** Identification of idiopathic nephrotic syndrome (INS)-specific extracellular vesicles (EVs)-microRNAs (miRNAs). (A) Venn diagram of overlapping miRNAs among the three datasets shows two miRNAs expressed in patients with INS compared to healthy volunteers (HVs) and idiopathic membranous nephropathy (IMN) subjects. (B) Fold change (FC) and p-values of two miRNAs, whose expression levels were up- or down-regulated in patients with INS compared to HVs and IMN. **Figure S5.** Extracellular vesicles (EVs)-microRNAs (miRNAs) from patients with idiopathic membranous nephropathy with clinical remission (IMN-W) and healthy volunteers (HVs). (A) Heat map showing z-scores of EVs-miRNAs from patients with IMN-W (n = 9) and HVs (n = 20) with 21 upregulated (yellow) and 23 downregulated (blue) miRNAs. (B) Fold change (FC) and p-values of top 10 miRNAs showed differential expression in patients with IMN-W and HVs. Figure S6. Extracellular vesicles (EVs)-microRNAs (miRNAs) from patients with idiopathic membranous nephropathy without clinical remission (IMN-R) and healthy volunteers (HVs). (A) Heat map showing z-scores of EVs-miRNAs from patients with IMN without clinical remission (IMN-R) (n = 10) and HVs (n = 20) with 37 upregulated (yellow) and 40 downregulated (blue) miRNAs. (B) Fold change (FC) and p-values of top 10 miRNAs showed differential expression in patients with IMN-R and HVs.

## Data Availability

As the study involved human participants, the data cannot be made freely available in the manuscript nor a public repository because of ethical restrictions. However, the data are available from Soonchunhyang University Hospital for researchers who meet the criteria for access to confidential data. Interested researchers can send data access requests to the corresponding author (KY, Doh, SH. Kwon).

## Abbreviations

IMN: Idiopathic membranous nephropathy
IMN-R: IMN with refractory response
IMN-W: IMN with well response
INS: Idiopathic nephrotic syndrome
PLA2R: Anti-phospholipase A2 receptor
EVs: Extracellular vesicles
miRNA: MicroRNA
AKI: Acute kidney injury
HV: Healthy volunteer
PCR: Polymerase chain reaction
qPCR: Quantitative PCR
NGS: Next-generation sequencing
FDR: False discovery rate
KEGG: Kyoto Encyclopedia of Genes
GO: Gene Ontology
snRNA: Small nuclear RNA
snoRNA: Small nucleolar RNA
TGF-β: Transforming growth factor-β
FoxO: Forkhead homobox type O
