# Supplementary material for: Circulating miRNAs in extracellular vesicles related to treatment response in patients with idiopathic membranous nephropathy
Source: J Transl Med. 2022 May 14;20:224. doi: 10.1186/s12967-022-03430-7 (PMC9107687; doi:10.1186/s12967-022-03430-7)
Supplement: Supplementary file 1 — Additional file 1: Figure S1. Bioanalyzer analysis of the size distribution of RNA from EVs. (A) The Pico 6000 chip analyzed total EV RNA (< 6000 nucleotides). (B) The Small RNA chip analyzed small RNA of EV (< 200 nucleotides). Figure S2. Small RNA composition differences in circulating extracellular vesicles (EVs) by RNA sequencing. *P < 0.05 vs patients with idiopathic membranous nephropathy with clinical remission (IMN-W), †P < 0.05 vs healthy volunteers (HVs). Figure S3. Performance of IMN-specific EV-miRNAs for the discrimination of IMN and INS. Receiver operating curves show the distinguishing performance of three miRNAs (miRNA-1229-3p, miRNA-340-3p, and miRNA-99b-5p) that obtained from 19 patients with IMN and 21 patients with INS. Figure S4. Identification of idiopathic nephrotic syndrome (INS)-specific extracellular vesicles (EVs)-microRNAs (miRNAs). (A) Venn diagram of overlapping miRNAs among the three datasets shows two miRNAs expressed in patients with INS compared to healthy volunteers (HVs) and idiopathic membranous nephropathy (IMN) subjects. (B) Fold change (FC) and p-values of two miRNAs, whose expression levels were up- or down-regulated in patients with INS compared to HVs and IMN. Figure S5. Extracellular vesicles (EVs)-microRNAs (miRNAs) from patients with idiopathic membranous nephropathy with clinical remission (IMN-W) and healthy volunteers (HVs). (A) Heat map showing z-scores of EVs-miRNAs from patients with IMN-W (n = 9) and HVs (n = 20) with 21 upregulated (yellow) and 23 downregulated (blue) miRNAs. (B) Fold change (FC) and p-values of top 10 miRNAs showed differential expression in patients with IMN-W and HVs. Figure S6. Extracellular vesicles (EVs)-microRNAs (miRNAs) from patients with idiopathic membranous nephropathy without clinical remission (IMN-R) and healthy volunteers (HVs). (A) Heat map showing z-scores of EVs-miRNAs from patients with IMN without clinical remission (IMN-R) (n = 10) and HVs (n = 20) with 37 upregulated (yello [file 12967_2022_3430_MOESM1_ESM.pptx]

## Slide 1
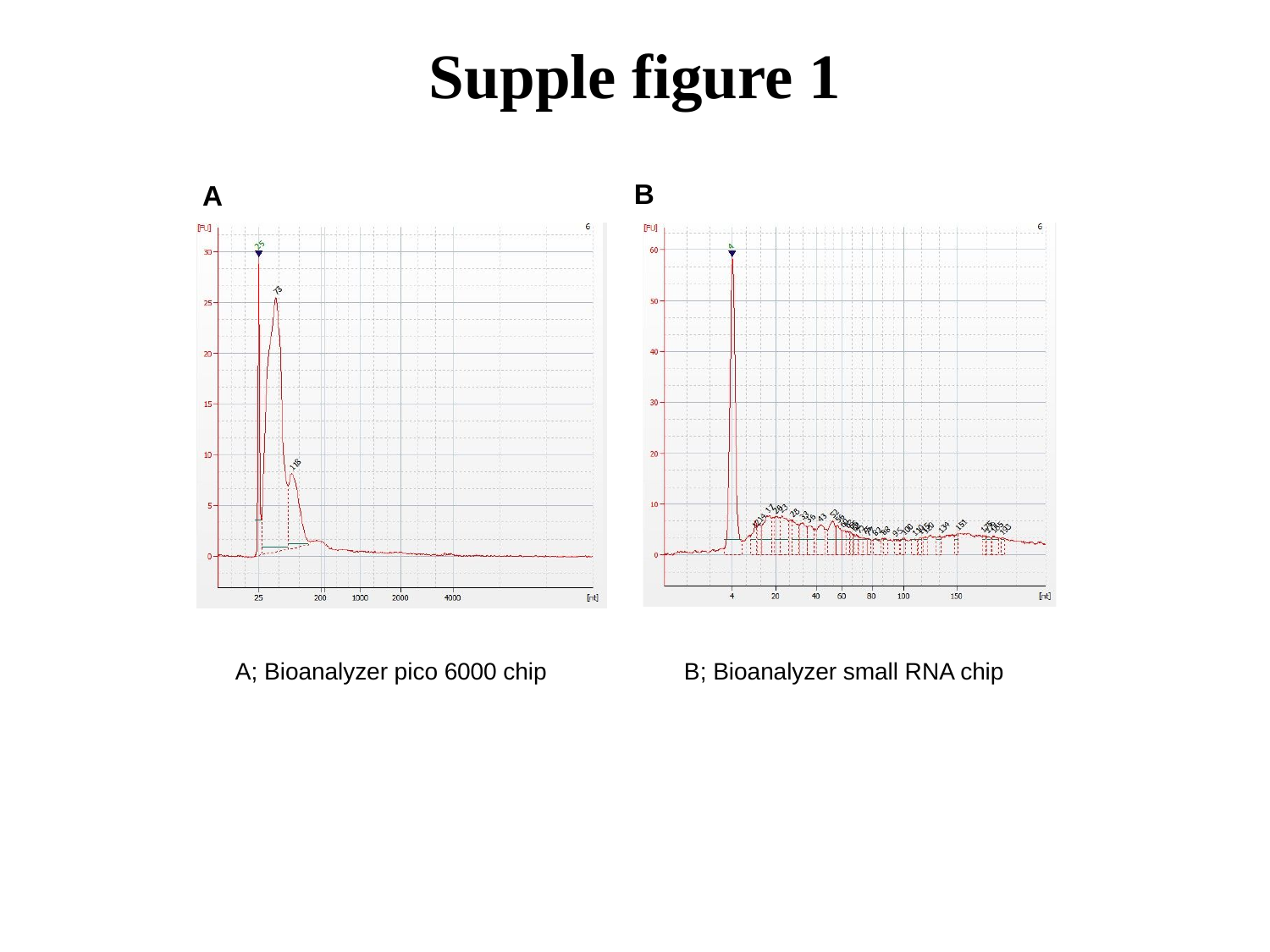

Supple figure 1
B
A
 A; Bioanalyzer pico 6000 chip B; Bioanalyzer small RNA chip

## Slide 2
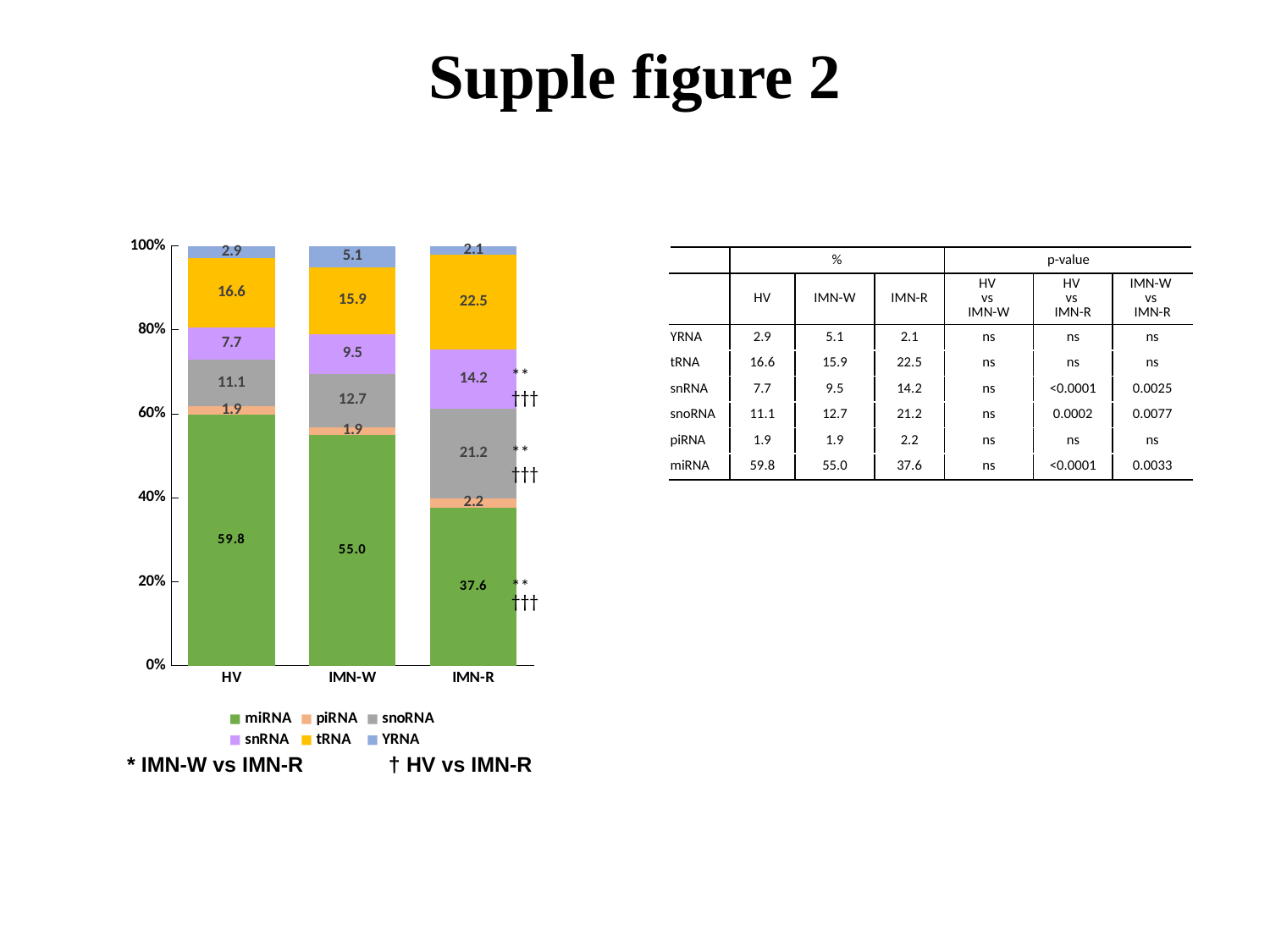

Supple figure 2
### Chart
| Category | miRNA | piRNA | snoRNA | snRNA | tRNA | YRNA |
|---|---|---|---|---|---|---|
| HV | 59.809543820716144 | 1.9494653734360732 | 11.141663275589208 | 7.656823319745699 | 16.60742708684026 | 2.874821760810563 |
| IMN-W | 55.02749004969064 | 1.8661205931797222 | 12.685388784126571 | 9.502403274273828 | 15.877736363657148 | 5.1120826112178985 |
| IMN-R | 37.631144300175876 | 2.218271076214951 | 21.2200712401219 | 14.198467570659787 | 22.496925681545427 | 2.136063262671536 || | % | | | p-value | | |
| --- | --- | --- | --- | --- | --- | --- |
| | HV | IMN-W | IMN-R | HV vs IMN-W | HV vs IMN-R | IMN-W vs IMN-R |
| YRNA | 2.9 | 5.1 | 2.1 | ns | ns | ns |
| tRNA | 16.6 | 15.9 | 22.5 | ns | ns | ns |
| snRNA | 7.7 | 9.5 | 14.2 | ns | <0.0001 | 0.0025 |
| snoRNA | 11.1 | 12.7 | 21.2 | ns | 0.0002 | 0.0077 |
| piRNA | 1.9 | 1.9 | 2.2 | ns | ns | ns |
| miRNA | 59.8 | 55.0 | 37.6 | ns | <0.0001 | 0.0033 |
**
†††
**
†††
**
†††
* IMN-W vs IMN-R
† HV vs IMN-R

## Slide 3
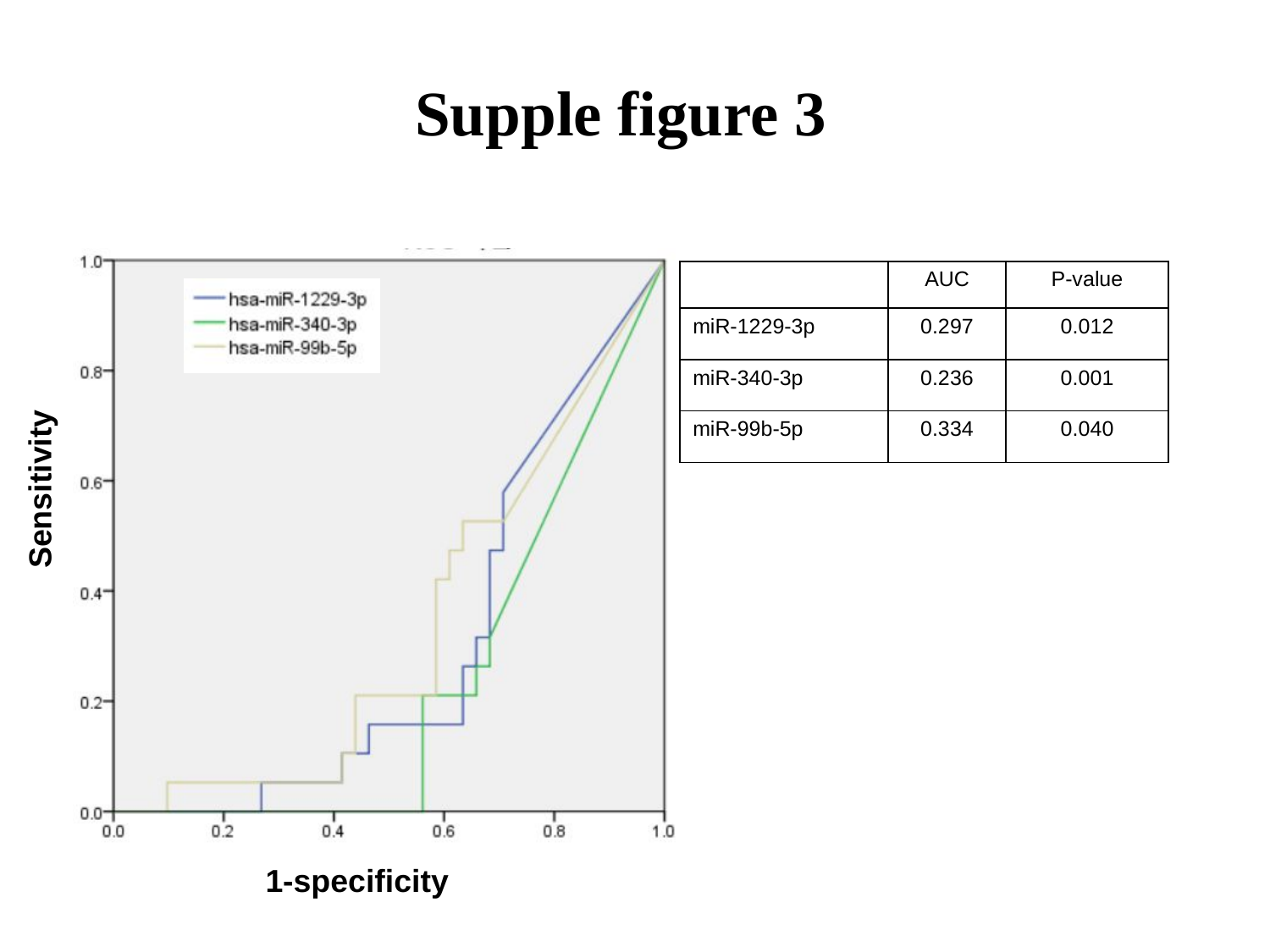

Supple figure 3
| | AUC | P-value |
| --- | --- | --- |
| miR-1229-3p | 0.297 | 0.012 |
| miR-340-3p | 0.236 | 0.001 |
| miR-99b-5p | 0.334 | 0.040 |
Sensitivity
1-specificity

## Slide 4
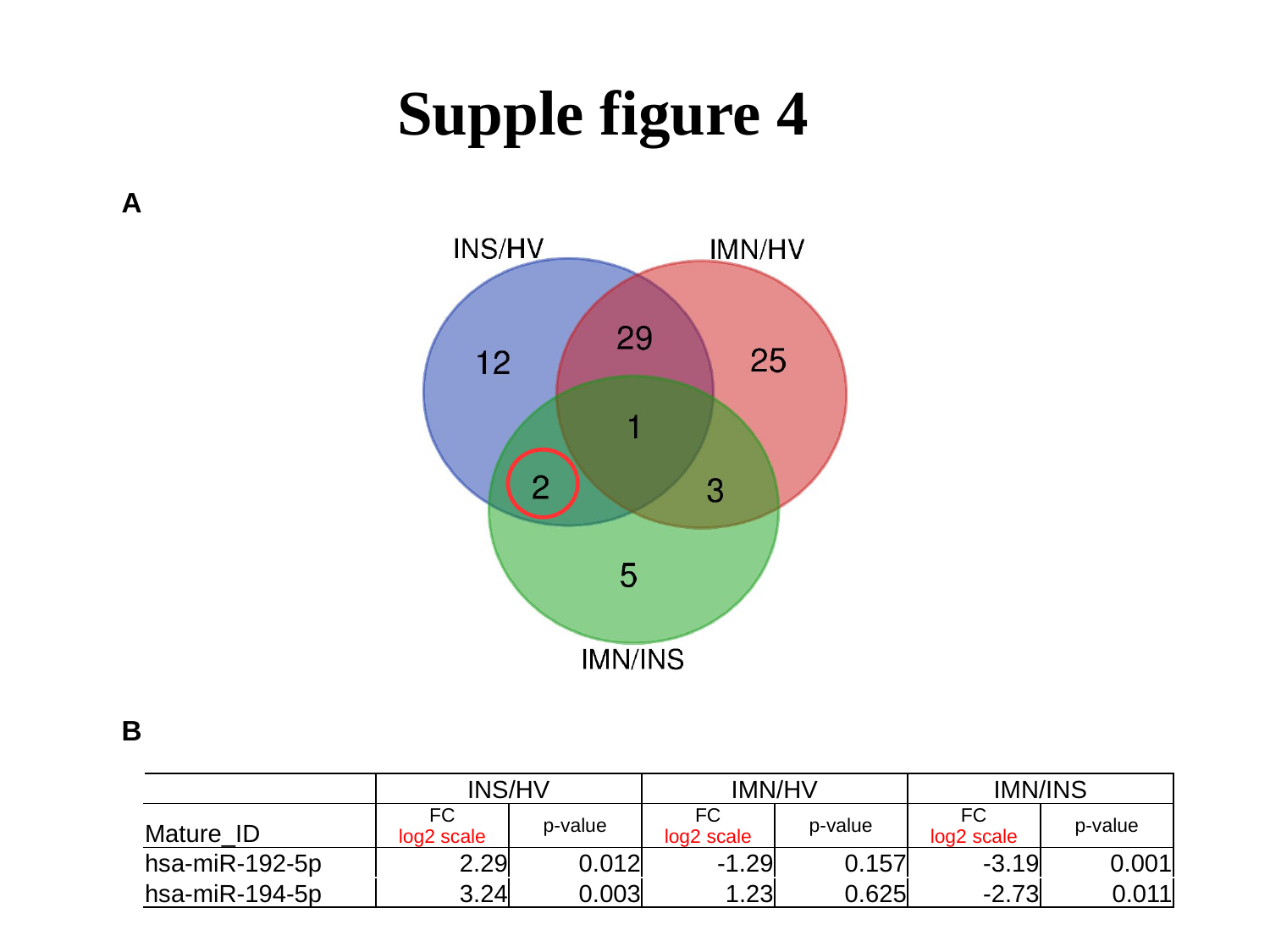

Supple figure 4
A
B
| | INS/HV | | IMN/HV | | IMN/INS | |
| --- | --- | --- | --- | --- | --- | --- |
| Mature\_ID | FC log2 scale | p-value | FC log2 scale | p-value | FC log2 scale | p-value |
| hsa-miR-192-5p | 2.29 | 0.012 | -1.29 | 0.157 | -3.19 | 0.001 |
| hsa-miR-194-5p | 3.24 | 0.003 | 1.23 | 0.625 | -2.73 | 0.011 |

## Slide 5
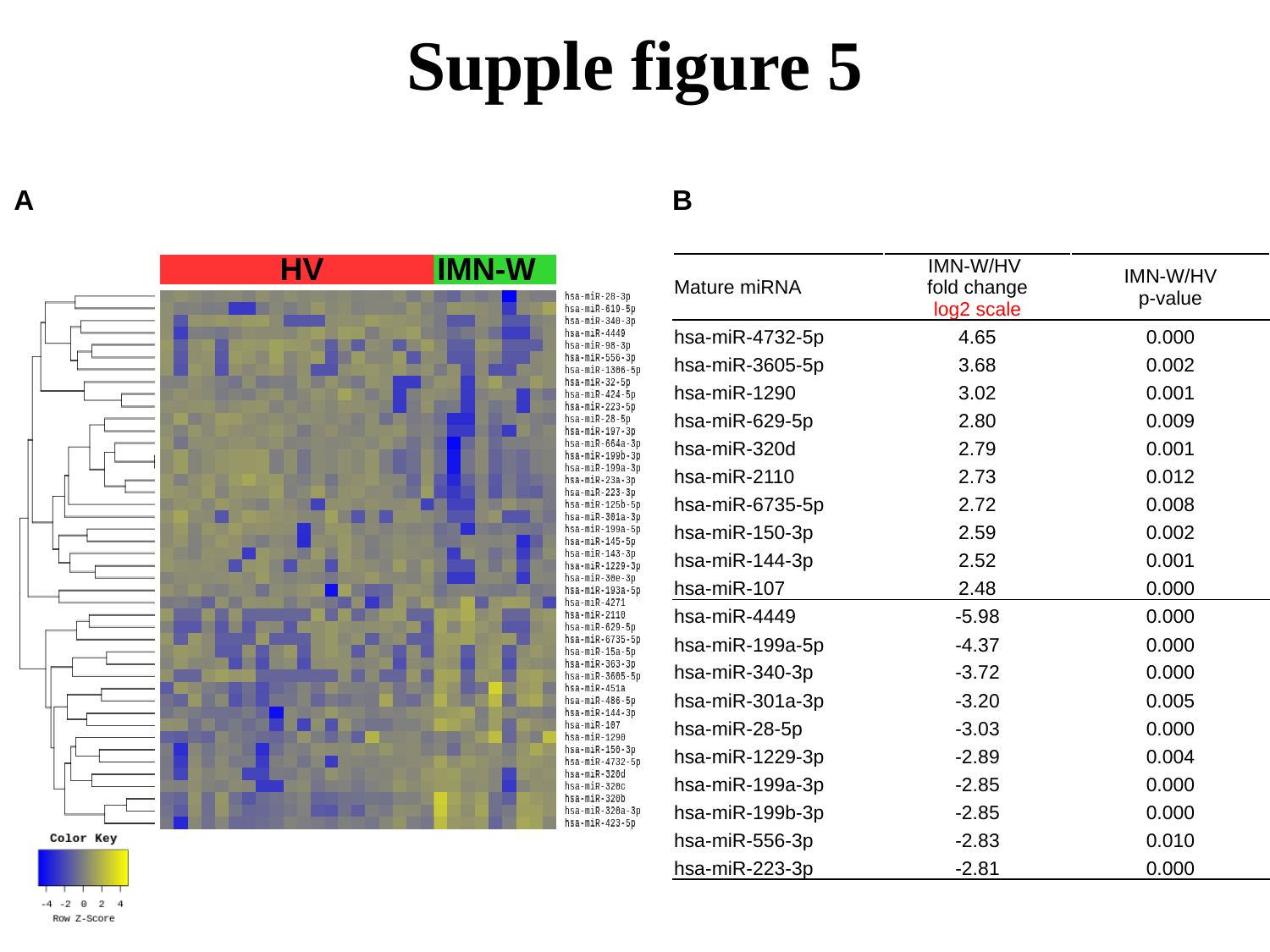

Supple figure 5
A
B
HV
IMN-W
| Mature miRNA | IMN-W/HV fold change log2 scale | IMN-W/HV p-value |
| --- | --- | --- |
| hsa-miR-4732-5p | 4.65 | 0.000 |
| hsa-miR-3605-5p | 3.68 | 0.002 |
| hsa-miR-1290 | 3.02 | 0.001 |
| hsa-miR-629-5p | 2.80 | 0.009 |
| hsa-miR-320d | 2.79 | 0.001 |
| hsa-miR-2110 | 2.73 | 0.012 |
| hsa-miR-6735-5p | 2.72 | 0.008 |
| hsa-miR-150-3p | 2.59 | 0.002 |
| hsa-miR-144-3p | 2.52 | 0.001 |
| hsa-miR-107 | 2.48 | 0.000 |
| hsa-miR-4449 | -5.98 | 0.000 |
| hsa-miR-199a-5p | -4.37 | 0.000 |
| hsa-miR-340-3p | -3.72 | 0.000 |
| hsa-miR-301a-3p | -3.20 | 0.005 |
| hsa-miR-28-5p | -3.03 | 0.000 |
| hsa-miR-1229-3p | -2.89 | 0.004 |
| hsa-miR-199a-3p | -2.85 | 0.000 |
| hsa-miR-199b-3p | -2.85 | 0.000 |
| hsa-miR-556-3p | -2.83 | 0.010 |
| hsa-miR-223-3p | -2.81 | 0.000 |

## Slide 6
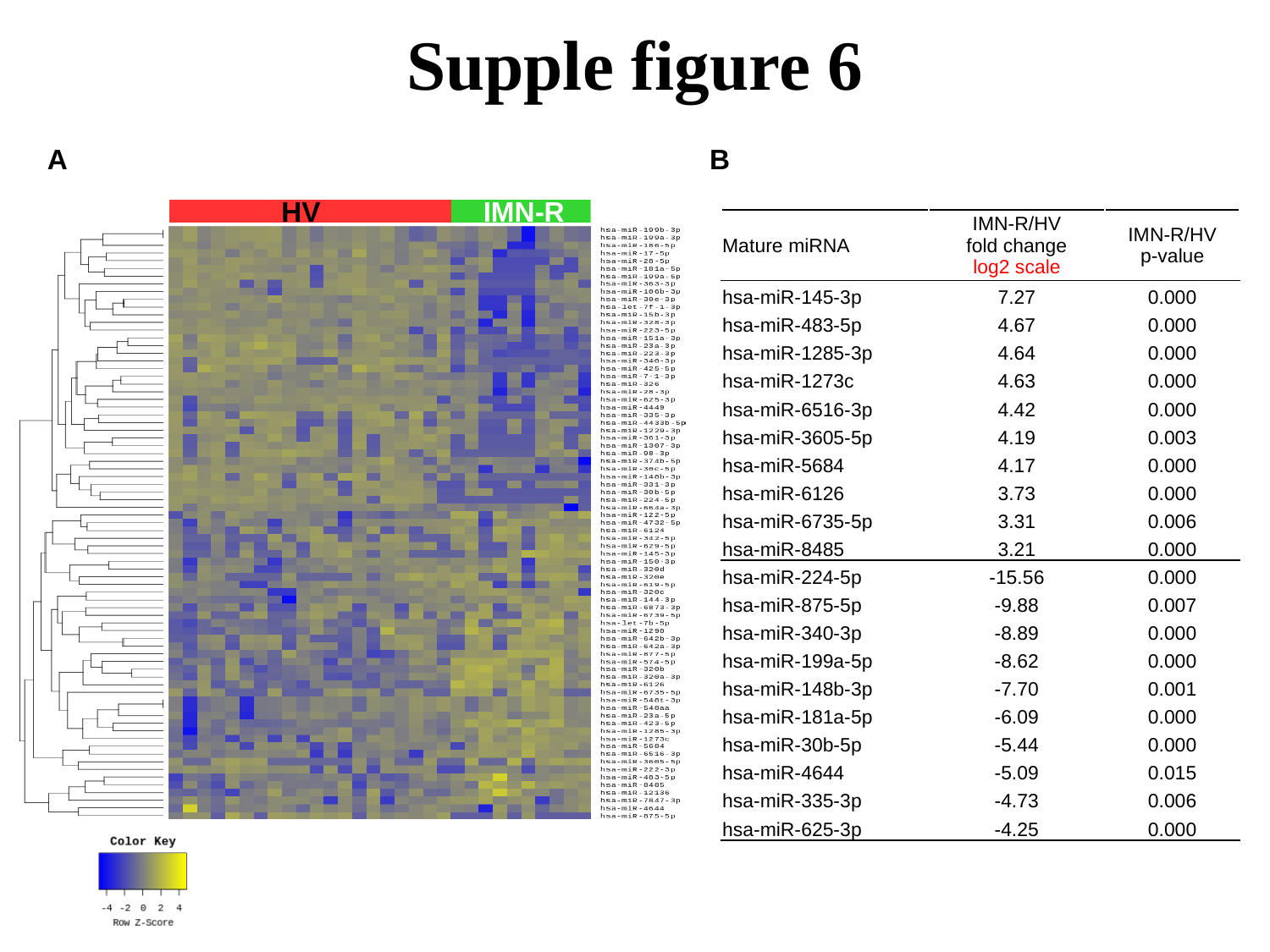

Supple figure 6
A
B
HV
IMN-R
| Mature miRNA | IMN-R/HV fold change log2 scale | IMN-R/HV p-value |
| --- | --- | --- |
| hsa-miR-145-3p | 7.27 | 0.000 |
| hsa-miR-483-5p | 4.67 | 0.000 |
| hsa-miR-1285-3p | 4.64 | 0.000 |
| hsa-miR-1273c | 4.63 | 0.000 |
| hsa-miR-6516-3p | 4.42 | 0.000 |
| hsa-miR-3605-5p | 4.19 | 0.003 |
| hsa-miR-5684 | 4.17 | 0.000 |
| hsa-miR-6126 | 3.73 | 0.000 |
| hsa-miR-6735-5p | 3.31 | 0.006 |
| hsa-miR-8485 | 3.21 | 0.000 |
| hsa-miR-224-5p | -15.56 | 0.000 |
| hsa-miR-875-5p | -9.88 | 0.007 |
| hsa-miR-340-3p | -8.89 | 0.000 |
| hsa-miR-199a-5p | -8.62 | 0.000 |
| hsa-miR-148b-3p | -7.70 | 0.001 |
| hsa-miR-181a-5p | -6.09 | 0.000 |
| hsa-miR-30b-5p | -5.44 | 0.000 |
| hsa-miR-4644 | -5.09 | 0.015 |
| hsa-miR-335-3p | -4.73 | 0.006 |
| hsa-miR-625-3p | -4.25 | 0.000 |
IMN-R
